# Regarding Transplantation of Hairy Skin

**Published:** 1913-02

**Authors:** 


Regarding Transplantation of Hairy Skin.—Carl Lauenstein, Hamburg (*Zent. fur Chir.*, September 7, 1912). A man who had tried every known method of specialism, as well as quackery, to obtain a growth of hair on his bald head, prevailed on Dr. Lauenstein to attempt the transplantation of a part of the well-covered scalp of another, whose consent was presumably bought, to the head of the first. In March, 1912, the exchange was made, using the whole skin. The transplant of hairy scalp, in area 10 cm. by 5 cm., broke down by septic necrosis on the head of the bald individual, whereas, the bald transplant was fairly successful on the head of the other.

